# JAG: A Computational Tool to Evaluate the Role of Gene-Sets in Complex Traits

**DOI:** 10.3390/genes6020238

**Published:** 2015-05-14

**Authors:** Esther S. Lips, Maarten Kooyman, Christiaan de Leeuw, Danielle Posthuma

**Affiliations:** 1 Department of Complex Trait Genetics, Neuroscience Campus Amsterdam, VU University & VU Medical Center, Amsterdam 1081HV, The Netherlands; E-Mail: e.s.lips@vu.nl; 2 Netherlands Bioinformatics Centre, Geert de Grooteplein 28, Nijmegen 6525 GA, The Netherlands; E-Mail: kooyman@gmail.com; 3 Delft Bioinformatics Lab, Delft University of Technology, Delft 2600GA, The Netherlands; 4 Radboud University Nijmegen, Nijmegen 6525EC, The Netherlands; E-Mail: c.deleeuw@science.ru.nl

**Keywords:** gene-set analysis, pathway analysis, biological pathway, genetic association, GWAS

## Abstract

Gene-set analysis has been proposed as a powerful tool to deal with the highly polygenic architecture of complex traits, as well as with the small effect sizes typically found in GWAS studies for complex traits. We developed a tool, Joint Association of Genetic variants (JAG), which can be applied to Genome Wide Association (GWA) data and tests for the joint effect of all single nucleotide polymorphisms (SNPs) located in a user-specified set of genes or biological pathway. JAG assigns SNPs to genes and incorporates self-contained and/or competitive tests for gene-set analysis. JAG uses permutation to evaluate gene-set significance, which implicitly controls for linkage disequilibrium, sample size, gene size, the number of SNPs per gene and the number of genes in the gene-set. We conducted a power analysis using the Wellcome Trust Case Control Consortium (WTCCC) Crohn’s disease data set and show that JAG correctly identifies validated gene-sets for Crohn’s disease and has more power than currently available tools for gene-set analysis. JAG is a powerful, novel tool for gene-set analysis, and can be freely downloaded from the CTG Lab website.

## 1. Introduction

The advent of genome-wide association (GWA) analysis has resulted in the identification of a large number of human disease genes and disease-related genetic variants for several traits such as type-2 diabetes, macular degeneration and Crohn’s disease [1,2,3,4]. At the same time, large scaled GWA studies with sample sizes ranging from 10,000 to 50,000 have shown that for many traits, such as educational attainment [5], schizophrenia [6], body mass index [7], and height [8], identification of trait-related variants tends to be less straightforward. These traits tend to be highly polygenic, with the estimated number of contributing genes in the hundreds or even thousands [5,7,8,9]. Given the current reasonably large sample sizes (e.g., nearly 250,000) [7], effect sizes of the majority of individual genetic variants that are yet to be identified are generally believed to be very small, difficult to detect, and of disputable etiological value [10].

Gene-set analysis has been proposed as an important tool in overcoming the “polygene-small effect” problem [11,12,13,14]. It reduces complexity, increases statistical power and has increased explanatory power compared to single gene or single SNP based analyses [15]. In a typical gene-set analysis, a test is conducted to evaluate the association of genetic variants located in a predefined set of genes with the trait under study. The gene-set analysis provides one test statistic (and one *p*-value) for the association of all genetic variants in the gene-set, and thus does not suffer from testing the multiple genetic variants in isolation. The pre-defined gene-set is typically chosen such that association of the gene-set with a trait can be directly interpreted in the context of a known function of the gene-set. For example, a gene-set can be constructed based on all genes that are known to be involved in the same biological pathway, on a set of genes that are co-expressed in a certain brain region, or on genes that are known to have a similar cellular function. Although gene-set analysis is sometimes also referred to as “pathway analysis”, when genes involved in biological pathways are grouped together for a single analysis, the term “gene-set analysis” is preferred to reflect the varying sources of defining gene-sets.

In recent years, several software tools for gene-set analysis have been proposed, such as GATES [16], ALIGATOR [12], the set-based test in PLINK [17], GENGEN [18] and GRASS [19], and see the review by Wang *et al.* [20]. These tools differ on several aspects such as the type of input data required (raw data *versus* summary statistics), using a self-contained or competitive test and the actual alternative hypothesis being tested [20].

The type of input data required by gene-set analysis tools is typically either summary statistics (*p*-values from a regular GWAS), or raw genotypes. While summary statistics are sometimes preferred for practical purposes (e.g., data sharing restrictions) and timing, Gui *et al.* [21] showed that gene-set tools that use raw data as input are generally more powerful than tools that rely on summary statistics.

Current gene-set tools also differ on whether they apply a so-called self-contained test or a competitive test [22,23]. In a self-contained test it is tested whether the genes in the gene-set are associated with the trait under investigation, without considering genes outside the gene-set. The null-hypothesis is that there is no association with the trait in any of the genes. The self-contained test is sensitive to spurious association, which thus needs to be taken into account for example in the data cleaning steps. In addition, for a highly heritable and polygenic trait the result of the self-contained test might be biased, as the null hypothesis strictly is not true. A competitive test [22,23] evaluates the association of a gene-set with a trait in the context of the associations of other gene-sets. The null hypothesis for a competitive test is that the selected gene-set is not more strongly associated to the trait than any other set of genes. The competitive test is not sensitive to spurious association due to population stratification, and is robust to the possibility of spurious associations due to polygenic signal in well-powered GWAS.

Available gene-set tools can also differ in the specific, alternative hypothesis being tested. For example, the hypothesis tested might be whether at least one genetic variant in the gene-set is associated with the trait (e.g., GATES and GENGEN), or whether *N* best *p*-values of genetic variants per gene in a gene-set are associated (e.g., GSA-SNP [24]). Some tools test whether there is an enrichment of genetic variants with a *p*-value below a certain threshold within the gene-set (e.g., ALIGATOR) or the other way around: tests whether the top *N* genetic variants from a regular GWAS are found more often than expected by chance in a certain gene-set. Some of these alternative hypotheses require more assumptions and decisions than others, as in determining *p*-value thresholds or setting a limit on the number of top genetic variants. These alternative hypotheses are not always made explicit, but obviously they will lead to different outcomes between different tools.

Currently available tools for gene-set analysis do not necessarily include both self-contained and/or competitive tests, do not always easily accommodate the use of custom gene-sets and some of them do not optimally make use of the multivariate evidence of association of all genetic variants in a gene-set. To allow stringent, easy, and powerful evaluation of the association of gene-sets with complex traits, we have developed an easy-to-use tool that can do all of the above: JAG (Joint Association of Genetic variants). JAG uses permutation to assess statistical significance and preferably requires raw genotypic data as input, but can also be applied to summary statistics. As permutations can be time-consuming, JAG includes features that facilitate the use of a cluster computer. JAG includes both self-contained and competitive test options, and also allows gene-based testing.

The performance of JAG is compared to other tools currently available for the self-contained gene-based and gene-set analysis. Comparison of competitive testing tools for gene-sets was not carried out as different tools test different null and alternative hypotheses rendering comparison of statistical properties uninformative. For the self-contained gene-set test, we compared the performance of our tool with the performance of the self-contained test available within PLINK’s set-based test [17], GATES-Simes [16] and GRASS [19]. We compared the performance of the self-contained gene based test in JAG with the self-contained gene-based test in VEGAS [25], SET-SCREEN [26] and GATES-Simes [16]. For all tools we conducted a Type I error analysis and a power analysis using the WTCCC Crohn’s disease dataset. We show that JAG correctly identifies previously validated gene-sets for Crohn’s disease and is more powerful than any of the other tools tested.

## 2. Implementation

**Figure 1:**
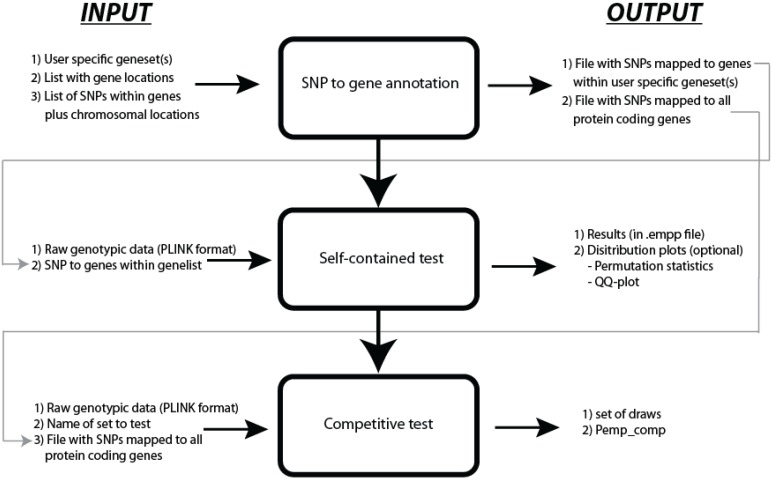
Workflow of JAG.

JAG is written in the Python programming language and depends on PLINK [17] and R [27]. JAG requires raw genotypic data as input files in PLINK binary format (.bim, .bed and .fam files) and an ASCII text file that contains the gene-set information. When summary statistics are used as input, JAG requires raw data from a reference population. JAG runs as a command line program on UNIX/Linux, Mac OS X and Windows operating systems. Typically, the use of JAG consists of three steps: SNP-to-gene annotation, self-contained testing, and competitive testing (see Figure 1). The first step may also be conducted outside of JAG.

### 2.1. SNP to Gene Annotation

As part of the pre-processing, JAG provides the option to map SNPs to the genes within the user-specified gene-sets. This option is supplied to simplify the pre-processing part of the analysis for the user. Within the SNP to gene annotation function, JAG maps the SNPs to genes, based on the transcription start site (TSS) and transcription end site (TES) of these genes. In addition, it is possible to map SNPs to genes within a user-specified ‘proximate regulatory region’ around the gene boundaries with a maximum of 100 Kb up- and/or downstream from the TSS and/or TES. To facilitate this SNP to gene mapping, a list of protein-coding genes is supplied together with their chromosomal coordinates for both HG18/NCBI build 36.3 and HG19/NCBI build 37.3. In addition, a list with validated SNPs located within 100kb from at least one TSS or TES of a protein-coding gene for both dbSNP130 (HG18) as dbSNP135 (HG19) is provided (based on the UCSC database). The user is not obliged to use this pre-processing function and is not restricted to use the supplied mapping files. Instead, the user can use custom based SNP to gene mapping.

### 2.2. Self-Contained Gene(-Set) Test

The self-contained test in JAG tests the null hypothesis that a gene or gene-set is not associated with the trait under investigation, given LD structure of included SNPs. This test can be conducted on both case/control information and quantitative traits. JAG constructs a multivariate gene(-set) test-statistic by summing the –log_10_ of the *p*-values from all the SNPs in the gene(-set)(s). These SNP-level based *p*-values are obtained by performing an association analysis in PLINK [17], which is invoked by JAG. The significance of a single gene-set is evaluated by a permutation procedure in which a large number of permutations (*i.e*., 1000 or 10,000) are conducted, resulting in one empirical *p*-value (*P*_EMP_SC_) per gene-set. This *P*_EMP SC_ is calculated by dividing the number of times the Σ−log_10_(*P*) from the permuted datasets exceeds or equals the Σ-log_10_(*P*) from the original dataset by the number of permutations run (Equation (1)).

(1) $$P_{EMP\_SC}=\frac{\sum_{n=1}^{N_{PERMS}}(\Sigma -log_{10}{\left(P\right)}_{PERMS}>\Sigma -log_{10}{\left(P\right)}_{REAL})}{N_{PERMS}}$$

Based on the empirical distribution generated for the self-contained test, the number of effective SNPs per gene-set is calculated. This number can be used for competitive testing when matching randomly drawn gene-sets on the effective number of SNPs. The effective number of SNPs is calculated as follows: under the null hypothesis of no association, –log_10_(*P*) is distributed as 1/(2ln(10)) = 0.217 times a χ2 with 2 degrees of freedom. If all M SNPs in a gene-set are independent from each other, then Σ–log_10_(*P*) has a mean of (0.217)(2M) and a variance of (0.217)^2^(4M) = 0.189M. The calculation of the effective number of SNPs (nEff) is based on the empirical distribution of the Σ−log_10_(*P*) under the null hypothesis of no association of the *N* permutations [9], and is defined as

(2) $$M_{eff}=\;\frac{M_{obs}[\sigma_{exp,\Sigma -log_{10}\left(P\right)}^{2}|SNPs_{ind}]}{\sigma_{exp,\Sigma -log_{10}\left(P\right)}}=\frac{M_{obs}\left[{\left(0.217\right)}^{2}\left(4M_{obs}\right)\right]}{\sigma_{exp,\Sigma -log_{10}\left(P\right)}}=\frac{0.189M_{obs}^{2}}{\sigma_{exp,\Sigma -log_{10}\left(P\right)}}$$

where the expected mean and variance are calculated on the number of SNPs that are summed to obtain the Σ−log_10_(*P*) per gene-set. A larger variance of the observed distribution than expected indicates dependency (*i.e*., due to LD) between included SNPs [14]. When summary statistics are used, a reference population (e.g., HapMap or 1kGenomes) is used with a simulated phenotype under the null hypothesis of no association in order to determine the distribution of the test statistic under the null hypothesis.

JAG includes both linear and logistic regression models for association analysis, in which covariates can be included. For example, it is possible to include principal components as a covariate in order to correct for population stratification. These principal components can be calculated outside of JAG, e.g., in EIGENSTRAT [28]. In addition to the gene-set based test, JAG has the option to conduct a self-contained test on the single-gene level. JAG can also provide QQ-plots for the distribution of the *p*-values of the genotyped SNPs, together with plots of the empirical distribution of the original and permuted test-statistic per gene-set. These plots are generated in R (http://cran.r-project.org/).

### 2.3. Competitive Test

The competitive test evaluates whether a certain gene-set of interest is more associated with the trait under investigation than a randomly generated, matched set of genes. This type of test is more robust to spurious association due to population stratification, and we strongly advise to interpret results from competitive testing only (conditional on statistically significant self-contained testing). JAG includes the option to perform a competitive test on a single gene-set by drawing, matched randomly generated sets of genes/genetic variants and testing these for association. Subsequently, the *P*_EMP_SC_ of the original gene-set is evaluated against the empirical *p*-values for the self-contained tests on the random draws, by calculating the number of times that the *P*_EMP_SC_ for the random draws is lower than the *P*_EMP_SC_ for the original gene-set (Equation (3)), which provides the competitive *p*-value (*P*_EMP_COMP_).

(3) $$P_{EMP\_COMP}=\;\frac{\sum_{n=1}^{N_{DRAWS}}(P_{EMP\_SC\_RANDOM}<\;\;P_{EMP\_SC\_ORIG})}{N_{DRAWS}}$$

Ideally, the randomly generated sets should contain the same number of genes and same number of SNPs per gene as the gene-set of interest. However, it is usually not feasible to fulfill both conditions when generating multiple random gene-sets, as that would severely limit the pool of SNPs and genes from which random sets can be drawn without creating a strong dependency between the drawn sets. Such dependency would result in biased competitive *p*-values. Therefore, JAG allows to create randomly generated control sets matched either for the number of genes in the original gene-set or for the effective number of SNPs in the original gene-set. In addition, random sets can optionally be drawn from different SNP-pools, for example including only intergenic SNPs, or only intragenic SNPs or a combination of those, each testing different alternative hypotheses.

### 2.4. Application and Performance of JAG: WTCCC Crohn’s Disease Data

To compare the performance of our tool with other tools we used the WTCCC Crohn’s Disease (CD) dataset, a set of 97 genes previously identified for Crohn’s disease and a set of 53 “benchmark gene-sets”. We have downloaded the Crohn’s Disease (cases), NBS (controls) and 1958 Birth Cohort (controls) data from the WTCCC website (http://www.wtccc.org.uk). All samples were genotyped on the Affymetrix 500k array. We conducted quality control on these datasets by removing the SNPs and individuals as recommended by WTCCC, followed by a quality control as described in the protocol from Anderson *et al.* [29]. We removed the SNPs with a minor allele frequency (MAF) <0.01, Hardy-Weinberg equilibrium (HWE) *p* < 0.00001, a missing rate > 5% and 3321 SNPs that were assigned to chromosome 0 by WTCCC. This resulted in a total of 399,906 SNPs for 1694 cases and 2917 controls after quality control.

### 2.5. Genes and Canonical Pathways for Gene-Set Analysis

A list of 97 genes that were previously reported to be associated with Crohn’s disease was obtained from references [3] and [21] (see Supplementary Table S1).

A total of 880 canonical pathways were downloaded from the MSigDB database (http://www.broadinstitute.org/gsea/msigdb/index.jsp). From this set of gene-sets, we selected only those gene-sets that contained between 10 and 300 genes as the use of gene-sets with less than 10 or more than 300 genes is not recommended for gene-set analyses [30]. This resulted in a total of 860 sets available for gene-set analysis comprising 6387 unique genes.

To construct gene-sets that are potentially associated with CD from these 860 canonical pathways, we performed an overrepresentation analysis in GeneTrial [31] on 97 genes that were previously reported as genes associated with CD [3]. GeneTrial calculated for each of the 860 gene-sets whether the number of CD associated genes observed in the gene-set is significantly higher than expected, by returning an FDR corrected *p*-value for the association of the gene-set. According to this analysis, 53 (out of 860) gene-sets showed significant overrepresentation of CD-associated genes, and these were used as “benchmark gene-sets” when evaluating the power of JAG in comparison to that of other gene-based or gene-set based tools. Details of these 53 gene-sets are given in Supplementary Table S2.

### 2.6. Comparison of JAG Performance with other Tools

We compared the performance of the self-contained gene and gene-set test of JAG with other tools that incorporate a self-contained test and use raw data as input.

The performance of the self-contained gene-set test of JAG is compared with the performance of PLINK (v1.07), GATES (v2.5) and GRASS (v0.1). We used the default setting for each of these tools unless other settings were needed to accommodate comparison. For GRASS, we used the default settings except for the gene definition: using “abs” to map SNPs within the absolute genome location of the gene and a “dist” of “0” to indicate that SNPs are mapped to a gene only in case a SNP is located within the physical location of a gene (within TSS and TES). In addition, we conducted analyses in PLINK with a set of parameters that makes PLINK more comparable with JAG (--set-r2 1; --set-p 1; --set-max 99999).

To compare the performance of the self-contained gene-based test in JAG we used VEGAS (v0.8.27), GATES (v2.5) and SETSCREEN (option in PLINK v1.07) with default settings. The performance of each of these tools is tested via a Type I error analysis and a power analysis.

### 2.7. Type I Error Analysis

For the Type I error analysis, 100 permutation of the phenotype (of the WTCCC CD dataset) were created under the null hypothesis. Subsequently, we evaluated the 6387 MSigDB genes for the gene-based tools, and the 860 canonical gene-sets in the gene-set based tools (as described above). The Type I error-rate was calculated per dataset as the proportion of genotyped genes or gene-sets that showed a nominal *p*-value < 0.05. The reported Type I error rate is the average Type I error rate over the 100 datasets.

### 2.8. Relative Power Indication Analysis

To evaluate the relative power of each tool, we calculated the proportion of detected benchmark gene-sets for each gene-set tool and the proportion of detected previously associated Crohn’s disease genes for each gene-based tool. We performed 10,000 permutations to construct a reliable nominal *p*-value and defined a gene or gene-sets as associated when showing a *p* < 0.05 after FDR correction. The relative power of each method to detect the benchmark gene-sets is reported via a hypergeometric test (conducted in R), which tests the deviation of the observed number of significant (benchmark) gene-sets from expected for each method with random sampling from the total number of (benchmark) gene-sets. With this method, we determined the relative performance of JAG compared to other tools in detecting association of these benchmark sets.

## 3. Results and Discussion

### 3.1. Type I Error Analysis—Gene-Set Based Test

We conducted a gene-set based Type I error analysis for JAG, GATES-Simes, GRASS, and PLINK. For PLINK, we performed an analysis with both the default settings as well as PLINK with settings similar to JAG. Table 1 shows the results of the Type I error analysis over the 860 MSigDB pathways and 100 datasets created under the null hypothesis. All tools show a Type I error rate in the range 0.0397–0.0541, and GATES-Simes was the most conservative in detecting false positives (0.0397).

**Table 1:** Type I error rates for the compared gene-set tools.

Algorithm	Type I Error
JAG	0.0541
GATES-Simes	0.0397
GRASS	0.0513
PLINK (default setting)	0.0475
PLINK (JAG settings)	0.0535

### 3.2. Power Analysis—Gene-Set Based Test

We compared the power to detect benchmark gene-sets of the self-contained gene-set test within JAG with the self-contained gene-set test available in VEGAS, SETSCREEN and GATES. Results of this power analyses are shown in Table 2. All tools had sufficient power to detect “benchmark gene-sets” in the WTCCC CD dataset. However, JAG detected the largest number of significant gene-sets after FDR correction (24 out of 860) and the largest number of benchmark gene-sets (9 out of 53). PLINK with JAG-like settings showed a very similar result with respect to the number of significant gene-sets detected (23 out of 860) but a lower number of benchmark gene-sets (7 out of 53). PLINK with default settings detected the smallest number of gene-sets, although these were all flagged as benchmark gene-sets.

**Table 2:** Power analysis for the different gene-set tools.

Algorithm	N Significant Gene-Sets (out of 860 (after FDR Correction))	N Significant Benchmark Gene-Sets (out of 53)	Hypergeometric Test
JAG	24	9	4.16E−6
GATES-Simes	14	6	8.48E−5
GRASS	10	5	1.47E−4
PLINK (default settings)	3	3	2.22E−4
PLINK (JAG settings)	23	7	2.59E−4

**Figure 2:**
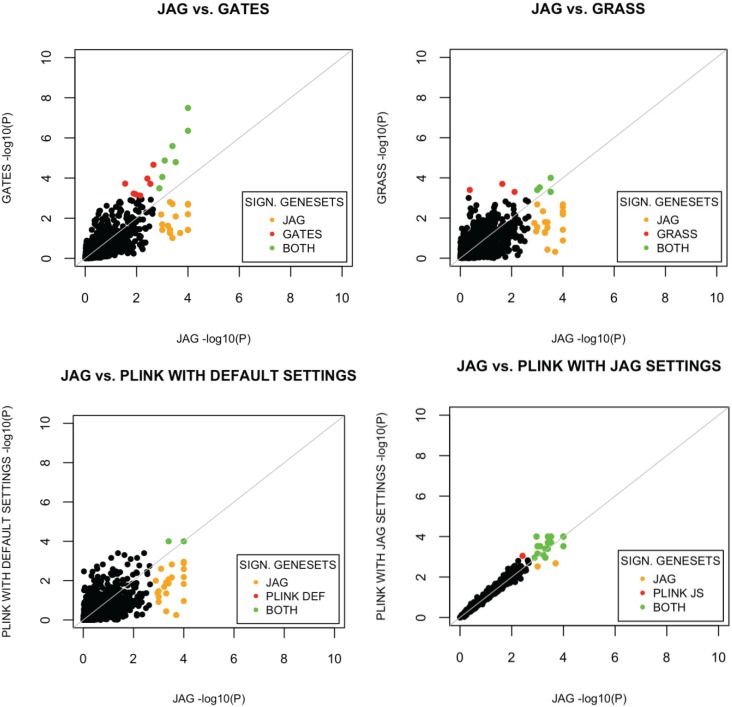
Comparison of results from JAG’s gene-set based test results with other gene-set based tools. The −log_10_ transformed *p*-values per gene-set are plotted. Yellow dots indicate gene-sets showing significance in JAG only. Red dots indicate gene-sets with statistically significant association in the comparison tool, but not in JAG. Green dots indicate gene-sets showing significance in both tools.

We also investigated the overlap in significant gene-sets detected by each method *versus* JAG. The results are visualized in the plots in Figure 2 (more details available in Supplementary Table S3). The *p*-values obtained with PLINK using JAG-like settings showed a high correlation with JAG, whereas results from the other methods were less correlated with JAG, which partly reflects different underlying null hypotheses of these methods.

### 3.3. Type I Error Analysis—Gene-Based Test

The performance of the self-contained gene-based test of JAG was compared with the gene-set test in VEGAS, GATES-Simes and SETSCREEN. The results of the Type I error analysis for gene-based test are presented in Table 3. The tools showed a Type I error rate in the range of 0.0438 and 0.0526, with SETSCREEN having the lowest Type I error rate and GATES the highest. JAG showed correct false positive rates of 0.0503.

**Table 3:** Type I error rates for gene-based test.

Algorithm	Type I Error
JAG	0.0503
GATES-Simes	0.0526
SETSCREEN	0.0438
VEGAS	0.0505

### 3.4. Power Analysis—Gene-Based Test

A power analysis was conducted over the 6387 genes from the MSigDB gene-sets (see Table 4).

**Figure 3:**
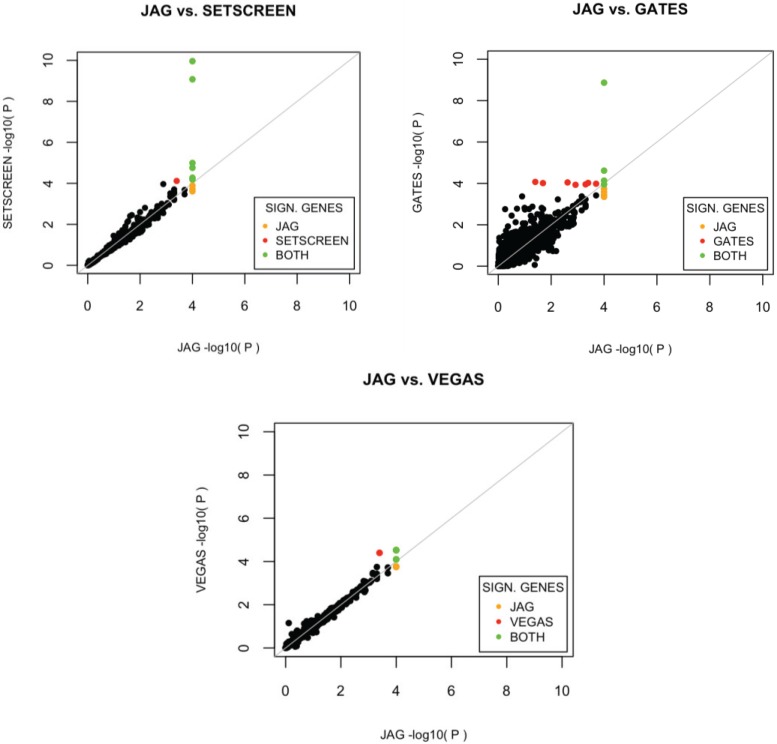
Comparison of results from JAG’s gene-based test results with other gene-based tools. The −log_10_ transformed *p*-values per gene-set are plotted. Yellow dots indicate gene-sets showing significance in JAG only. Red dots indicate gene-sets with statistical significant association in the comparison tool, but not in JAG. Green dots indicate gene-sets showing significance in both tools.

GATES performed best in the gene-based analysis (Hypergeometric test *p* = 2.30E−8), followed by JAG (Hypergeometric test *p* = 6.82E−7). In Figure 3 the overlap in significant genes for JAG against the other tools is depicted and shows that GATES and JAG show the lowest correlation in *p*-values per gene, but are closest in power to detect benchmark genes. See Supplementary Table S4 for additional details on the genes detected per method.

**Table 4:** Power analysis for gene-based test.

	Nsig_FDR	Nsig_prev	Hypergeometric Test
JAG	11 (4479)	4 (32)	6.82E−7
GATES-Simes	13 (4517)	5 (34)	2.30E−8
SETSCREEN	8 (4481)	3 (32)	1.81E−5
VEGAS	11 (6099) *	4 (52)	1.48E−6

* Note that VEGAS included ~1600 additional genes compared to the other tools. This difference occurs due to the mapping of SNPs to genes, which occurs separately within each of the tools. In the mapping function of VEGAS SNPs are mapped to genes via PLINK by using PLINK’s parameters “--from-kb” and “--to-kb”. This mapping provides comparable results to other tools, as long as there are SNPs located within the gene boundaries (TSS and TES). However, in case there are no SNPs located within the gene boundaries, PLINK assigns the SNP nearest to the TSS as well as the SNP nearest to the TES to the gene. Nsig_FDR = N significant genes after FDR correction (N total genes tested). Nsig_prev = N significant genes of previously associated genes (N total mapped genes of previously associated list).

## 4. Conclusions

Gene-set analysis has become a powerful tool to translate GWAS findings into biological mechanisms of disease. Our tool for gene-set analysis is powerful and easy to use. Here, we showed that using a standard dataset JAG correctly identifies validated gene-sets and has more power than currently available tools for gene-set analysis. JAG captures the added value of multiple associated SNPs in a gene-set, conditional on LD structure, and will be most powerful when multiple independent SNPs in a gene-set show at least some evidence of association, but is less likely to yield a significant gene-set *p*-value when a single SNP is associated moderately to the trait. The algorithms implemented in JAG were previously successfully applied to intelligence [13] and two large GWAS datasets of schizophrenia implicating three synaptic gene-sets [6,14], providing novel biological insights for these traits. In the current study, we systematically compared the performance of JAG’s self-contained test -on gene-set level as well as on gene based level- to other self-contained gene-set analysis tools that use raw genotype data as input, but differ in their algorithm to test for the alternative hypothesis. For example, some algorithms test whether a truncated set of SNPs (based on *p*-value) is associated with a trait (e.g., GATES and PLINK with default settings) or whether the average *p*-value of all SNPs in a set (PLINK with JAG settings) is significantly lower than expected under the null. In practice, this could mean that in the situation that only one SNP is associated with a trait, an algorithm that tests whether at least one SNP in the gene-set is associated may provide a significant *p*-value for the gene-set, whereas an algorithm that tests whether there is multivariate evidence for association in the gene-set may not. These differences in alternative hypotheses between available algorithms should be kept in mind when interpreting differences in outcomes. On the basis of the Crohn’s disease dataset that was used to compare the different tools, JAG showed the best performance on gene-set level. A more extensive comparison of performance of different tools across a wide number of scenarios is beyond the scope of the current study, yet would shed light on the circumstances under which different tools provide different outcomes. For example it would be informative to know how the size of the gene-set and the distribution of polygenic effects influence type I and type II errors for different tools. Also, we like to note that the selection of benchmark gene sets against which performance of tools can be tested, is critical. At present, there are very few diseases for which sets of genes have shown causative effects, which complicate the creation of standardized benchmarks.

Although JAG offers the option of both self-contained and competitive testing, we like to point out that in the context of polygenic traits, the results of competitive testing should be interpreted and not the self-contained results. This is because the polygenic nature of a trait may easily result in a statistically significant self-contained while the competitive testing scheme allows interpretation in the context of polygenic background influences.

In summary, JAG is a freely available open source tool for gene-set and gene-based analysis, which has already been successfully applied by us as well as others [6,13,14]. JAG has high flexibility, high statistical power and is easy to use. Our tool uses raw genotype data or summary statistics as input and permutation in order to obtain an accurate empirical *p*-value. Furthermore, JAG includes an option to perform snp-to-gene annotation, allows for both self-contained and competitive testing with or without including covariates and provides intuitive plots for visual interpretation of results.

## Supplementary Files

Supplementary File 1

## References

[1] Visscher P.M., Brown M.A., McCarthy M.I., Yang J. (2012). Five years of GWAS discovery. Am. J. Hum. Genet.

[2] Sullivan P.F., Daly M.J., O’Donovan M. (2012). Genetic architectures of psychiatric disorders: The emerging picture and its implications. Nat. Rev. Genet.

[3] Franke A., McGovern D.P., Barrett J.C., Wang K., Radford-Smith G.L., Ahmad T., Lees C.W., Balschun T., Lee J., Roberts R. (2010). Genome-wide meta-analysis increases to 71 the number of confirmed Crohn's disease susceptibility loci. Nat. Genet.

[4] Voight B.F., Scott L.J., Steinthorsdottir V., Morris A.P., Dina C., Welch R.P., Zeggini E., Huth C., Aulchenko Y.S., Thorleifsson G. (2010). Twelve type 2 diabetes susceptibility loci identified through large-scale association analysis. Nat. Genet.

[5] Rietveld C.A., Medland S.E., Derringer J., Yang J., Esko T., Martin N.W., Westra H.J., Shakhbazov K., Abellaoui A., Agrawal A. (2013). GWAS of 126,559 individuals identifies genetic variants associated with educational attainment. Science.

[6] Ripke S., O’Dushlaine C., Chambert K., Moran J.L., Kahler A.K., Akterin S., Bergen S.E., Collins A.L., Crowley J.J., Fromer M. (2013). Genome-wide association analysis identifies 13 new risk loci for schizophrenia. Nat. Genet.

[7] Speliotes E.K., Willer C.J., Berndt S.I., Monda K.L., Thorleifsson G., Jackson A.U., Lango Allen H., Lindgren C.M., Luan J., Mägi R. (2010). Association analyses of 249,796 individuals reveal 18 new loci associated with body mass index. Nat. Genet.

[8] Lango Allen H., Estrada K., Lettre G., Berndt S.I., Weedon M.N., Rivadeneira F., Willer C.J., Jackson A.U., Vedantam S., Raychaudhuri S. (2010). Hundreds of variants clustered in genomic loci and biological pathways affect human height. Nature.

[9] Purcell S.M., Wray N.R., Stone J.L., Visscher P.M., O’Donovan M.C., Sullivan P.M., Sklar P., International Schizophrenia Consortium (2009). Common polygenic variation contributes to risk of schizophrenia and bipolar disorder. Nature.

[10] Visscher P.M. (2008). Sizing up human height variation. Nat. Genet.

[11] Torkamani A., Topol E.J., Schork N.J. (2008). Pathway analysis of seven common diseases assessed by genome-wide association. Genomics.

[12] Holmans P., Green E.K., Pahwa J.S., Ferreira M.A., Purcell S.M., Sklar P., Owen M.J., O’Donovan M.C., Craddock N., Wellcome Trust Case-Control Consortium (2009). Gene ontology analysis of GWA study data sets provides insights into the biology of bipolar disorder. Am. J. Hum. Genet.

[13] Ruano D., Abecasis G.R., Glaser B., Lips E.S., Cornelisse L.N., de Jong A.P., Evans E.M., Davey Smith G., Timpson N.J., Smi A.B. (2010). Functional gene group analysis reveals a role of synaptic heterotrimeric G proteins in cognitive ability. Am. J. Hum. Genet.

[14] Lips E.S., Cornelisse L.N., Toonen R.F., Min J.L., Hultman C.M., Holmans P.A., O’Donovan M.C., Purcell S.M., Smit A.B., International Schizophrenia Consortium (2012). Functional gene group analysis identifies synaptic gene groups as risk factor for schizophrenia. Mol. Psychiatry.

[15] Khatri P., Sirota M., Butte A.J. (2012). Ten years of pathway analysis: current approaches and outstanding challenges. PLoS Comput. Biol..

[16] Li M.X., Gui H.S., Kwan J.S., Sham P.C. (2011). GATES: A rapid and powerful gene-based association test using extended Simes procedure. Am. J. Hum. Genet.

[17] Purcell S., Neale B., Todd-Brown K., Thomas L., Ferreira M.A., Bender D., Maller J., Sklar P., de Bakker P.I., Daly M.J. (2007). PLINK: A tool set for whole-genome association and population-based linkage analyses. Am. J. Hum. Genet.

[18] Wang K., Li M., Bucan M. (2007). Pathway-based approaches for analysis of genomewide association studies. Am. J. Hum. Genet.

[19] Chen L.S., Hutter C.M., Potter J.D., Liu Y., Prentice R.L., Peters U., Hsu L. (2010). Insights into colon cancer etiology via a regularized approach to gene set analysis of GWAS data. Am. J. Hum. Genet.

[20] Wang K., Li M., Hakonarson H. (2010). Analysing biological pathways in genome-wide association studies. Nat. Rev. Genet.

[21] Gui H., Li M., Sham P.C., Cherny S.S. (2011). Comparisons of seven algorithms for pathway analysis using the WTCCC Crohn’s Disease dataset. BMC Res. Notes.

[22] Tian L., Greenberg S.A., Kong S.W., Altschuler J., Kohane I.S., Park P.J. (2005). Discovering statistically significant pathways in expression profiling studies. Proc. Natl. Acad. Sci. USA.

[23] Goeman J.J., Buhlmann P. (2007). Analyzing gene expression data in terms of gene sets: Methodological issues. Bioinformatics.

[24] Guo Y.F., Li J., Chen Y., Zhang L.S., Deng H.W. (2009). A new permutation strategy of pathway-based approach for genome-wide association study. BMC Bioinform..

[25] Liu J.Z., McRae A.F., Nyholt D.R., Medland S.E., Wray N.R., Brown K.M., Hayward N.K., Montgomery G.W., Visscher P.M., AMFS Investigators (2010). A versatile gene-based test for genome-wide association studies. Am. J. Hum. Genet.

[26] Moskvina V., O’Dushlaine C., Purcell S., Craddock N., Holmans P., O’Donovan M.C. (2011). Evaluation of an approximation method for assessment of overall significance of multiple-dependent tests in a genomewide association study. Genet Epidemiol..

[27] R Development Core Team (2011). R: A Language and Environment for Statistical Computing.

[28] Price A.L., Patterson N.J., Plenge R.M., Weinblatt M.E., Shadick N.A., Reich D. (2006). Principal components analysis corrects for stratification in genome-wide association studies. Nat. Genet.

[29] Anderson C.A., Pettersson F.H., Clarke G.M., Cardon L.R., Morris A.P., Zondervan K.T. (2010). Data quality control in genetic case-control association studies. Nat. Protoc..

[30] Ramanan V.K., Shen L., Moore J.H., Saykin A.J. (2012). Pathway analysis of genomic data: Concepts, methods, and prospects for future development. Trends Genet.

[31] Keller A., Backes C., Al-Awadhi M., Gerasch A., Kuntzer J., Kohlbacher O., Kaufmann M., Lenhof H.P. (2008). GeneTrailExpress: A web-based pipeline for the statistical evaluation of microarray experiments. BMC Bioinform..

